# HNF1B polymorphism influences the prognosis of endometrial cancer patients: a cohort study

**DOI:** 10.1186/s12885-015-1246-5

**Published:** 2015-04-07

**Authors:** Vincenzo Dario Mandato, Enrico Farnetti, Federica Torricelli, Martino Abrate, Bruno Casali, Gino Ciarlini, Debora Pirillo, Maria Carolina Gelli, Davide Nicoli, Mario Grassi, Giovanni Battista LA Sala, Stefano Palomba

**Affiliations:** 1 Unit of Obstetrics and Gynecology, IRCCS-Arcispedale Santa Maria Nuova, Reggio Emilia, Italy; 2 Laboratory of Molecular Biology, IRCCS-Arcispedale Santa Maria Nuova, Reggio Emilia, Italy; 3 Unit of Pathology, IRCCS-Arcispedale Santa Maria Nuova, Reggio Emilia, Italy; 4 Department of Brain and Behavioral Science, Medical and Genomics Statistics Unit, University of Pavia, Pavia, Italy; 5 University of Modena and Reggio Emilia, Modena, Italy; 6 Unit of Gynecologic Oncology, IRCCS-CROB, Rionero in Vulture (Potenza), Potenza, Italy

**Keywords:** Endometrial cancer, Rs4430796, HNF1B, SNP, Adjuvant therapy, Survival

## Abstract

**Background:**

HNF1B (formerly known as TCF2) gene encodes for a transcription factor that regulates gene expression involved in normal mesodermal and endodermal developments. A close association between rs4430796 polymorphism of HNF1B gene and decreased endometrial cancer (EC) risk has been demonstrated. The aim of the current study was to test the hypothesis that rs4430796 polymorphism can influence the prognosis of EC patients.

**Methods:**

Retrospective cohort study. Clinical and pathological data were extrapolated and genotypes were assessed on formalin-fixed and paraffin-embedded non-tumour tissues. The influence of patients’ genotype on overall survival and progression free survival were our main outcome measures.

**Results:**

A total of 191 EC patients were included in the final analysis. Overall survival differed significantly (*P* = 0.003) among genotypes. At multivariate analysis, a significant (*P* < 0.05) effect on overall survival was detected for FIGO stage, and rs4430796 polymorphism of HNF1B gene. After grouping EC patients according to adjuvant treatment, rs4430796 polymorphism resulted significantly (*P* < 0.001) related to overall survival only in subjects who received radiotherapy plus chemotherapy. A significant (*P* = 0.014) interaction between rs4430796 polymorphism and chemo-radiotherapy was also detected. Finally, only a trend (*P* = 0.090) towards significance was observed for rs4430796 polymorphism effect on progression free survival.

**Conclusions:**

rs4430796 polymorphism of HNF1B gene influences independently the prognosis of EC patients with a potential effect on tumor chemo-sensitivity.

## Background

Several genes in the sex steroid hormone metabolism pathway have been investigated for polymorphic variants that predispose to endometrial cancer (EC). Single nucleotide polymorphisms (SNPs) in these genes are associated with EC risk [1]. For example, *CYP19A1* (aromatase) genetic variation influenced susceptibility to EC, particularly among older and obese patients [1]. However, very little part of the genetic risk can be explained by these SNPs.

Hepatocyte nuclear factor-1beta (HNF1B) (formerly known as TCF2) gene encodes for a transcription factor that regulates gene expression involved in normal mesodermal and endodermal developments [2]. HNF1B protein exists in three isoforms: isoforms A and B, which act as transcriptional activators, and isoform C, which acts as a transcriptional repressor [2]. HNF1B regulates gene expression necessary for normal mesodermal and endodermal development, and is expressed in numerous tissues. The loss of function in HNF1B has been reported in genetic syndromes such as Mayer-Rokitansky-Kuster-Hauser [3-5] and Prune Belly syndrome [6]. In addition, the oncogenic role of HNF1B has been reported in several cancers such as renal, colorectal, gastric, pancreatic, and ovarian cancer. HNF1B overexpression has been reported to be a biomarker of ovarian clear-cell carcinoma (CCC), of its probable precursor, endometriosis and of EC [6-8]. HNF1B can be considered an oncogene [6-8] and plays a role in the origin of CCC not only in ovary but also in uterus [9,10]. Moreover, HNF1B expression was also investigated to classify the several EC histotypes [11].

A genome-wide association study (GWAS) identified a SNP (G allele of rs4430796) in HNF1B associated with decreased EC risk in women of European background [12]. In 2012, the Population Architecture using Genomics and Epidemiology (PAGE) study [13] confirmed that HNF1B SNP was associated with a decreased risk of EC in the populations of two independent studies [14-16]. The associations were observed across multiple racial/ethnic groups and similar associations were seen for both type I and type II ECs and across all categories of body mass index (BMI), parity, oral contraceptives use, menopausal hormone use, and smoking status [17].

More recently, in order to search additional common genetic variants, a further GWAS was conducted in EC populations participating in studies of the Epidemiology of Endometrial Cancer Consortium [18]. Previous data on G allele of rs4430796 were confirmed and no new SNPs were found [18]. Moreover, analysis of several lymphocyte-derived gene expression datasets indicated a significant association between rs4430796 genotype and HNF1B expression [12]. HNF1B expression may regulate the process of tumorigenesis [10] and the biological behavior of EC such as chemoresistance.

Despite several studies reported a relationship between rs4430796 and EC risk, no data is available in literature about the relationship between rs4430796 and EC prognosis. Based on these considerations, the purpose of the current experimental study was to test hypothesis that rs4430796 polymorphism influences the prognosis in EC patients.

## Methods

### Ethics

The Provincial Ethical Committee of Reggio Emilia approved the study design and all patients provided written informed consent to use personal non-sensitive data at hospital admission.

### Study protocol

The study design followed the Strengthening the Reporting of Observational Studies in Epidemiology (STROBE) statement [19]. Clinical charts of EC patients treated and followed at the IRCCS-Santa Maria Nuova Hospital of Reggio Emilia (Italy) from 1997 to 2010 were checked for inclusion and exclusion criteria.

Tissue samples of the included patients were retrieved, prepared, and stored in the tissue storage system of the Department of Pathology. Genomic DNA extraction and purification, and rs4430796 genotyping was performed in the Laboratory of Molecular Biology.

Clinical, pathological and genetic data were recorded in an electronic separate, anonymous, password-protected database. All relevant data were extrapolated and used for final analysis.

The overall survival (OS) and the progression free survival (PFS) were considered the two main outcome measures to assess EC prognosis. The OS was computed as the time period from the date of surgery to either the date of death or last follow up, whichever occurred first, and the PFS was computed as the disease-free period from the date of surgery to the date of relapse or last follow up, whichever occurred first.

### Population

Patients with histological diagnosis of EC who received upfront surgery treatment were electively included in the protocol study.

Exclusion criteria were: inadequate EC treatment according to internal and international guidelines [20,21], neoadjuvant chemotherapy performed before surgery, an age less than 18 years, non-Caucasian ancestry, a follow-up length shorter than 6 months, inadequate follow-up according to internal guidelines, absence of written informed consent, diagnosis of a previous or concurrent cancer(s) and unavailable follow-up data.

An adequate treatment was considered as follows: total extrafascial hysterectomy (TEH) with bilateral salpingo-oophorectomy (BSO) was the standard staging procedure; whereas radical hysterectomy (RH) was performed only in stage II EC patients with gross cervical involvement; pelvic with/without paraaortic lymph node dissection were performed in case of Type II EC, myometrial invasion greater than 50 percent, large tumor (>2 cm in diameter) or filling the endometrial cavity; omentectomy, appendicectomy (for mucinous cancers), peritoneal biopsies, and maximal tumor debulking were always performed in case of type II EC patients.

Vaginal brachytherapy alone was administered to patients at stage IA G3 and IB G1 or G2 without negative prognostic factors. External beam radiotherapy plus vaginal brachytherapy was administered to patients at stage IA G3 and IB G1 and G2 with negative prognostic factors, to patients at stage IB G3 and to all patients at stage II, III and IV. Chemotherapy was administered to patients at stage III C and IV. In all cases, chemoradiotherapy consisted of paclitaxel 175 mg/m2 (P) and carboplatin AUC5 (C) on day 1 every three weeks, for a total of four to six cycles, and it was followed by external pelvic radiation therapy (1.8 Gy/d, d1-5) at a total dose of 45 Gy plus vaginal brachytherapy (3 × 5 Gy) [20,22].

A follow-up was defined “adequate” in case of adherence to the following schedule: type I EC at stage IA and grading G1/G2 - physical and gynecological examination, and transvaginal ultrasound every 6 months for the first 2 years, and then every 12 months for at least 3 years; type I EC at stage IB and/or any grade G3 tumour and any EC type II - physical and gynecological examination, and transvaginal ultrasound every 6 months for the first 5 years. Further investigations such as abdominal ultrasound, chest X-ray, computed tomography scan, and CA 125 serum level were performed if clinically indicated.

### Tissue samples, DNA extraction, sequencing and analysis of rs4430796-SNP

The same pathologist (M.C.G.) with long-time expertise in gynecological oncology reviewed all the histological samples to confirm formally the diagnosis. Then, she cut the formalin-fixed and paraffin-embedded tissue in order to obtain 5 μm-thick slices of non-tumor tissue for genomic test.

Two operators (E.F, B.C.) performed the genomic DNA extraction and purification using the BiOstic® FFPE Tissue DNA Isolation Kit (MoBio Laboratories, Inc., Carlsbad, CA, US) and quantification using Nanodrop 2000 (Thermo Fisher Scientific Inc., DE, US).

Rs4430796 genotyping was performed by LGC Genomics / KBioscience (Hertfordshire, UK) using KASP™ genotyping assay, which employs a competitive allele-specific protein chain reaction (PCR) (http://www.Kbioscience.co.uk).

The minor and major alleles of rs4430796 SNP were defined as A and G, respectively.

### Statistical analysis

For statistical analysis, R-2.15.1 software was used.

Associations between rs4430796 and clinical and pathological parameters were assessed by generalized linear models. Survival curves were plotted using the Kaplan–Meier method and analyzed using the log-rank test. Cox regression hazard model was used for multivariate analysis to assess the independent influence of rs4430796 SNP and other covariates on overall survival and cancer recurrence.

Results were presented as hazard ratio (HR) and 95% confidence interval (CI). To evaluate combined effects between polymorphism and clinical and pathological parameters, interaction hazard ratios (IHR) were calculated fitting Cox model. IHR compared the observed HR due to variables synergy to the expected HR obtained by multiplication of single effect of each variable when the other one is not involved (IHR = HR_11_/HR_01_*HR_10_). IHR = 1 indicated no synergy between variables, IHR <1 expressed a reduction of risk due to variables synergy, while IHR >1 denoted an increased risk.

## Results

After patients careful selection for inclusion and exclusion criteria, 191 EC patients were studied and included in the final analysis.

In all EC patients a complete resection of the disease was obtained.

In Table 1 the main clinical and pathological data of the studied population are summarized [23].

**Table 1:** **Main clinical data in overall EC population and in EC patients categorized according to rs4430794 genotypes**

Characteristic	Patients (n.)	rs4430796		*P*value
		GG (n. %)	GA/AA (n. %)	
	**191**	**57**	**134**	
**Age** ^1^	64.3 ± 10.6	64.9 ± 10.1	64.0 ± 10.8	
**Histological type**				
*Type I*	162	46 (28.4)	116 (71.6)	
*Type II* ^*2*^	29	11 (37.9)	18 (62.1)	0.304
**Figo stage** ^**2**^				
*I*	152	45 (29.6)	107 (70.4)	
*II*	12	4 (33.3)	8 (66.7)	
*III*	23	6 (26.1)	17 (73.9)	
*IV*	4	2 (50.0)	2 (50.0)	0.820
**Grading (for Type I)**				
*G1*	70	17 (24.3)	53 (75.7)	
*G2*	63	18 (28.6)	45 (71.4)	
*G3*	29	11 (37.9)	18 (62.1)	0.186
**Myometrial invasion**				
*<50%*	100	30 (30.0)	70 (70.0)	
*>50%*	91	27 (29.7)	64 (70.3)	0.960
**Lymph node metastasis (129 lymphadenectomy)**				
*No*	113	34 (30.1)	79 (69.9)	
*Yes*	16	4 (25.0)	12 (75.0)	0.677
**Adjuvant treatment**				
*None*	119	36 (27.9)	83 (64.3)	
*Radiotherapy*	42	12 (28.6)	30 (71.4)	
*Chemo-Radiotherapy*	30	9 (30.0)	21 (70.0)	0.926
**Radiotherapy (42 patients)**				
*External beam therapy*	2	1 (50.0)	1 (50.0)	
*Brachytherapy*	21	6 (28.6)	15 (71.4)	
*Combined*	19	5 (26.3)	14 (73.7)	0.615

^1^ Age (years) is shown as mean ± SD.

^2^ Staging and grading were done according to the 2009 International Federation of Gynecology and Obstetrics (FIGO) classifications [23]. Women treated before 2009 were restaged according to the 2009 FIGO classifications.

Total extrafascial hysterectomy was performed in 179 of 191 patients (93.7%), radical hyesterctomy was performed in 12 of 191 patients (6.3%), salpingo-oophorectomy was performed in 184 of 191 patients (96.3%), omentectomy was performed in 35 of 191 patients (18.3%), appendicectomy was performed in 9 of 191 patients (4.7%), pelvic lymphadenectomy was performed in 129 of 191 patients (67.5%), lombo-aortic lymphadenectomy was performed in 15 of 191 patients (7.9%).

After a median follow-up time of 64 months (range, 7 to 151 months), a total of 18 patients died (18/191, 9.4%). Twenty two patients (22/191, 11.5%) had a recurrence. Sixteen of 18 dead patients (88.9%) had a recurrence. Overall survival was 46 months (range, 7 to 136 months).

A allele frequency of rs4430796 SNP was 0.46. GG, AG and AA genotypes were found in 57 (57/191, 29.8%), 91 (91/191, 47.6%) and 43 (43/191, 22.5%) patients, respectively. That genotypic distribution of polymorphism resulted in Hardy-Weinberg equilibrium (*P =* 0.56).

**Figure 1:**
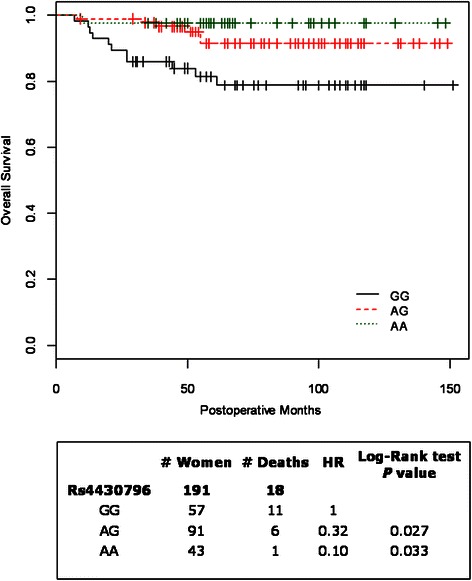
**Kaplan-Meier plot of estimate probability of overall survival in relation to three rs4430796 SNP genotypes.**

In Figure 1 is showed the estimate probability of OS according to three rs4430796 SNP genotypes. A significant (*P* = 0.003) difference among genotypes was observed, and the worst OS was observed for GG genotyped patients. Thus, further analyses were performed grouping AG and AA genotypes (*vs*. GG genotype) assuming dominant effect of A allele.

No difference in any clinical and pathological data was detected between GG and GA/AA stratified EC patients (Table 1).

The effect of the main clinical and pathological parameters on OS and PFS was tested using multivariate Cox model (Table 2). In the model data were adjusted only for age since in the present study EC FIGO grading could be applied only in a proportion of patients and patients’ performance status were similar in all cases since selected for “full surgery” and adjuvant therapy.

**Table 2:** **Effect of the main clinical and pathological parameters on overall survival and progression free survival using multivariate Cox models**

	Overall survival				Progression free survival		
	Multivariate Cox model				Multivariate Cox model		
	Women	Deaths	HR (95%CI)	*P*value*	Relapses	HR (95%CI)	*P*value*
**Histotype**	**191**	**18**			**22**		
*Type I*	162	9	1		13	1	
*Type II*	29	9	2.3 (0.56-9.22)	0.254	9	1.6 (0.51-5.38)	0.406
**Myometrial invasion**							
*<50%*	100	2	1		4	1	
*≥50%*	91	16	4.4 (0.74-26.07)	0.104	18	3.2 (0.82-12.10)	0.094
**FIGO stage**							
*I*	152	6	1		9	1	
*II*	12	3	5.9 (1.00-34.54)	0.050	4	5.4 (1.21-23.94)	0.027
*III*	23	6	6.1 (1.08-34.54)	0.041	6	4.1 (0.75-22.66)	0.102
*IV*	4	3	7.64 (0.84-69.49)	0.071	3	9.5 (1.16-77.7)	0.036
**Adjuvant treatment**							
*None*	119	6	1		10	1	
*Radiotherapy*	42	3	0.37 (0.07-1.95)	0.239	2	0.20 (0.04-1.05)	0.057
*Chemo-Radiotherapy*	30	9	0.94 (0.16-5.58)	0.947	10	0.65 (0.13-3.18)	0.598
**rs4430796**							
*GG*	57	11	1		10	1	
*AG + AA*	134	7	0.18 (0.06-0.56)	0.003	12	0.46 (0.18-1.13)	0.090

*CI* = confidence interval, *HR* = hazard ratio. * *P* values were calculated with Log-rank test and express statistical differences in overall survival and progression free survival.

Analysis showed a highly significant effect (*P* = 0.003) of rs4430796 polymorphism on OS while only a trend (*P* = 0.090) towards significance was observed for SNP effect on PFS. Between other parameters only FIGO stage maintained a slightly significant effect on OS and PFS after adjustment.

**Figure 2:**
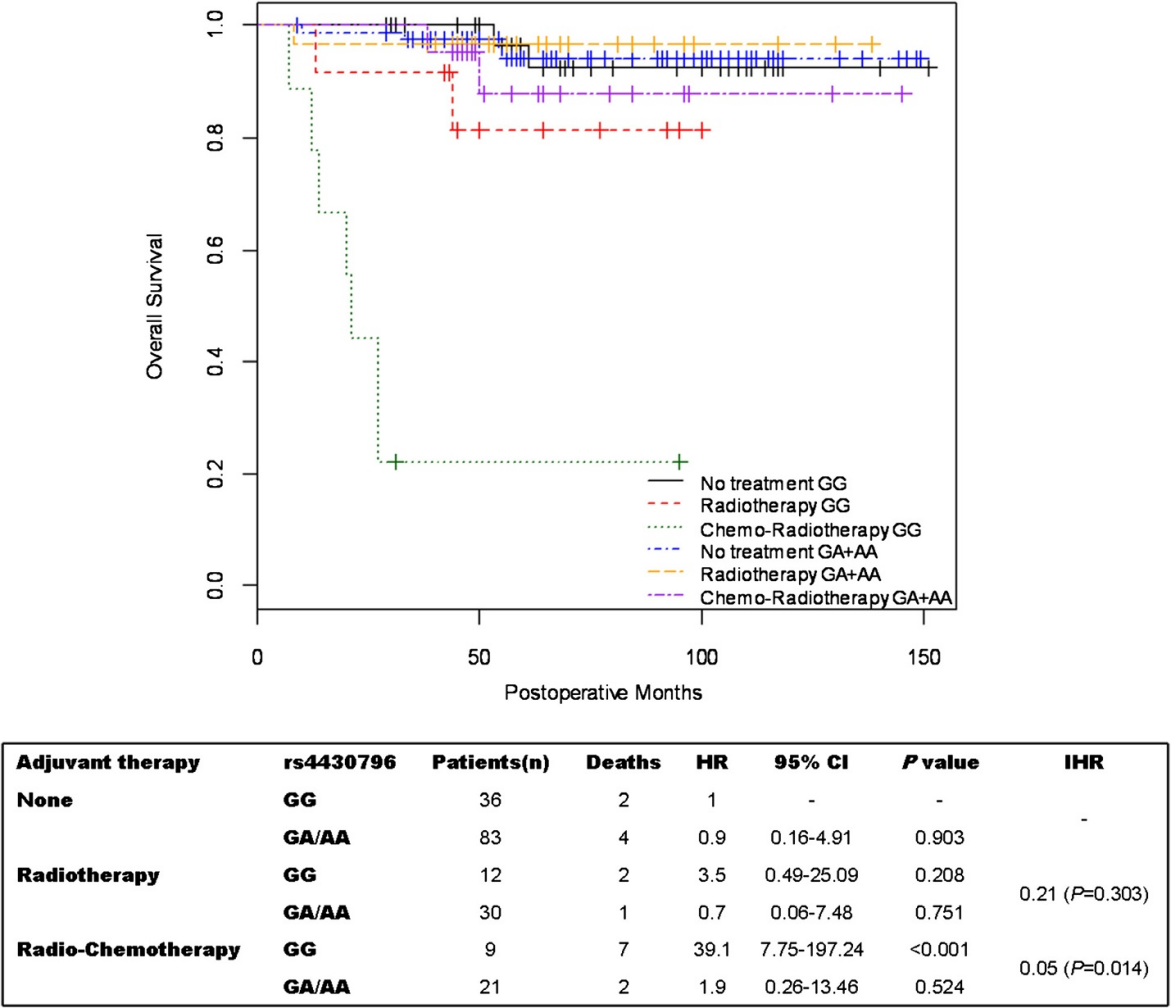
**Kaplan-Meier plot of estimate probability of overall survival comparing GG vs. AG + AA rs4430796 genotypes in EC patients classified according to adjuvant treatment.** Log-rank test *P* < 0.0001.

The effect of rs4430796 polymorphism on OS in EC patients according to adjuvant therapy is showed in Figure 2. No significant effect was detected in EC patients who did not receive adjuvant therapy and in those treated with radiotherapy (Figure 2). In EC patients who received radiotherapy plus chemotherapy a significant (*P* < 0.001) effect of rs4430796 polymorphism on OS was detected. A significant (*P* = 0.014) interaction between rs4430796 polymorphism and chemo-radiotherapy was also observed (Figure 2). A significantly (*P* < 0.001) worse OS was observed only in subjects with GG genotype who received radio-chemotherapy as adjuvant treatment (Figure 2).

In Figure 2, the estimate OS comparing different rs4430796 SNP genotypes in EC patients classified according to adjuvant therapy is showed. A significantly (*P* < 0.001) worse OS was observed only in subjects with GG genotype who received radio-chemotherapy as adjuvant treatment (Figure 2).

## Discussion

To our knowledge, this was the first study aimed to test the hypothesis that rs4430796 influences the OS of patients with EC. Current retrospective cohort study showed that the OS of EC patients varied significantly according to rs4430796 genotypes and at multivariate analysis rs4430796 SNP genotype resulted an independent risk factor for OS in EC patients. In particular, GG genotyped patients showed the worst OS.

In order to explore this influence, our population was studied according to adjuvant treatment. In EC patients treated with post-surgical radio-chemotherapy, subjects with GG genotype had a significant HR of 39.1 for death, even if a wide CI was observed. Of note, a significant interaction between rs4430796 polymorphism and treatment was detected showing that the impact on OS is different from that expected from the sum of the individual effects of the two variables. Those findings were not confirmed for EC patients who did not receive adjuvant therapy and for those who received radiotherapy alone, suggesting that rs4430796 polymorphism could play a crucial role in tumor sensitivity to chemotherapy. To this regard, no other study is available in literature and our study was the first to suggest that A allele of rs4430796 in EC patients undergoing chemo-radiotherapy might reduce the risk of death by increasing the response to therapy.

Well-known prognostic factors for EC include age, race, stage, grade, ploidy, depth of invasion, tumor size, receptor status, and cell type [24]. However, these multiple factors failed to give an accurate estimate of prognosis, whereas some genetic variants, such as rs4430796, may be in the next future a useful marker to predict, alone or in combination, the prognosis of EC patients.

Although many studies have investigated HNF1B in gynecological malignancies, such as CCC, showing its relationship with tumor cell survival [25] and tumor-associated thrombosis [6], few data are reported on its role in EC. A GWAS demonstrated that G allele of rs4430796 is associated with decreased EC risk in European population [12] and the PAGE study [13,14] subsequently confirmed those findings in two independent studies. The associations were observed irrespectively of several demographic, anthropometric, hormonal, voluptuary patients’ characteristics [17]. Finally, a more recent GWAS [18] confirmed previous finding [12] and no new SNPs were found. Recently, HNF1B was found to be overexpressed in EC [9-11], particularly in CC EC [26].

No data were found in literature explaining the role of rs4430796 genotype on HNF1B function but some contradictory studies supposed that rs4430796 polymorphism could influence HNF1B gene expression [12,27-30]. Spurdle *et al*. [12] analyzed several lymphocyte-derived gene expression datasets and identified significant associations between rs4430796 genotype and HNF1B expression in individuals of European ancestry. In the same population, a RNA sequencing experiment suggested that HNF1B expression in patients with different rs4430796 genotypes presented a trend of increase in read depth with increasing number of G alleles [12].

Recent and not always concordant studies analyzed the influence of HNF1B gene expression on chemosensitivity in ovarian cancer [27,28,30]. In particular, some studies, observing that ovarian CCC characterized by HNF1B overexpression were often chemo-resistant, suggested a possible effect of the level of HNF1B on chemosensitivity. It was demonstrated that shRNA mediated downregulation of HNF1B sensitizes ovarian cancer cells to cisplatin- or paclitaxel-mediated cytotoxicity, through inverse regulation of HSulf1 expression [27]. In 2014, an interesting mechanism of inhibition of cell death by HNF1B transcription factor was also proposed [28]. Specifically, it was demonstrated that chemoresistance that characterizes CCC might be due to aberrant retention of the G2 checkpoint of cell cycle induced by HNF1B overexpression [28]. In addition, HNF1B might induce this aberrant retention through the upregulation of CHK1 kinase, a protein that plays a pivotal role in the G2 checkpoint [28].

It was not possible for us to evaluate HNF1B gene expression in FFPE samples. The supposition of a potential biological model will require further experiments but, based on published studies, we suppose that the correlation that we observed between rs4430796 GG genotype and reduced OS in patients treated with chemotherapy could support the hypothesis that G allele reduces chemosensitivity through HNF1B overexpression. Identification of EC patients candidate for chemotherapy with rs4430796 GG genotype could be useful in the clinical practice in the next future to schedule a different chemotherapy and/or the administration of a sensitization agent. A recent study showed that the administration of CHK1 inhibitors to human cell line of CC EC treated with bleomicin was followed by an increase cell death rate [28]. Similarly, CHK1 inhibitors administration might decrease the chemoresistance to several drugs [28]. However, new agents that enhance the chemosensitivity should be investigated.

Unlike to HNF1B, ARID1A downexpression, mutation or loss of function is associated with chemoresistance and deep myometrial invasion in EC [31,32]. ARID1A is a tumor suppressor gene. Inactivating mutations of ARID1A and loss of its expression was found especially in endometrium-derived tumors, including ovarian CCC, ovarian endometrioid carcinomas and EC [33,34]. Both ARID1A and HNF1B play a role in the pathogenesis of ovarian CCC arose in endometriosis and both were proposed as new experimental markers in EC [8]. Usually, ARID1A mutation is an early event in neoplastic transformation and it is present both in carcinoma and preneoplastic tissue whilst HNF1B is usually found only in cancer tissue [8]. ARID1A mutations co-occur with mutations of PTEN and PIK3CA. Furthermore, PI3K pathway activity is regulated by ARID1A through phosphorylation of AKT [35]. Also AKT is involved in chemoresistance of EC cells, particularly Akt2 and Akt3 isoforms are involved in cell chemoresistance independently of the drug used for treatment [36,37]. Both AKT and HNF1B take part in the signaling pathway that regulates insulin secretion in pancreatic beta cells and their involvement in EC could suggest a possible role, not yet investigated, of these genes in the regulation of tumor cells glucose homeostasis [38,39] since abnormal glucose homeostasis is a well-known risk factor for EC type 1. However, despite a direct interaction between HNF1B and ARID1A/AKT was not yet clarified, aforementioned evidences suggest that a common pathway could be implicated in the pathogenesis and biological behavior of EC. Further studies need to elucidate these potential interactions from a biological and clinical point of view.

Current study presents issues of strength and limitation. The strength of our study regards the selection of a very homogeneous population of EC patients who received an upfront surgery with adequate treatment and follow-up length. The centralization of treatment, follow-up, and pathology review are further study strengths that ensured a uniformity of treatment, staging procedures, post-treatment monitoring and histological classification. Instead, in other available studies [13] the treatment and follow-up protocols widely varied [40]. Furthermore, our study has also important limitations. In particular, it might be underpowered because of the small cohort, and actual sample size might not be sufficient to detect a synergistic effect in a replicate study. However, despite the small sample size, the genotype distribution in our population comply with Hardy-Weinberg equilibrium indicating that identified significant association can be considered representative and not-biased by patients selection. Regarding to small sample size of this study, we did not manage to recruit a sufficient number of EC patients with advanced disease to request chemo-radiotherapy because most EC is diagnosed at early stage, and chemo-radiotherapy is commonly delivered in less than 10% of subjects [20-22]. Chemo-radiotherapy is generally administered to EC patients with positive lymph nodes (stage III) or distant metastases (stage IV) [20-22]. That population represents the 6-7% and 3% of EC patients, respectively of the overall EC population. In addition, the rate of 5-year survival for stage I disease is approximately 80-90%, for stage II, 70- 80%, and for stages III and IV, 20-60% [22]. Hence the mortality is also low.

## Conclusions

Current preliminary study demonstrates that the mortality of EC patients is significantly and independently influenced by rs4430796 polymorphism. This effect is probably exerted through tumor chemo-sensitivity. Further large multicenter studies need to confirm our results and to assess whether rs4430796 polymorphism could predict adjuvant treatment effectiveness in EC patients.

## Abbreviations

EC: Endometrial cancer
SNPs: Single nucleotide polymorphisms
CCC: Clear-cell carcinoma
GWAS: Genome-wide association study
PAGE: Population Architecture using Genomics and Epidemiology
PFS: Progression free survival
OS: Overall survival
STROBE: The Strengthening the Reporting of Observational Studies in Epidemiology
THE: Total extrafascial hysterectomy
BSO: Bilateral salpingo-oophorectomy
RH: Radical hysterectomy
HR: Hazard ratio
CI: Confidence interval
IHR: Interaction hazard ratios.
